# Experience of Intimate Partner Violence among Women in Sexual Unions: Is Supportive Attitude of Women towards Intimate Partner Violence a Correlate?

**DOI:** 10.3390/healthcare9050563

**Published:** 2021-05-11

**Authors:** Richard Gyan Aboagye, Joshua Okyere, Abdul-Aziz Seidu, John Elvis Hagan, Bright Opoku Ahinkorah

**Affiliations:** 1 School of Public Health, University of Health and Allied Sciences, PMB 31 Ho, Ghana; raboagye18@sph.uhas.edu.gh; 2 Department of Population and Health, University of Cape Coast, PMB TF0494 Cape Coast, Ghana; joshuaokyere54@gmail.com (J.O.); abdul-aziz.seidu@stu.ucc.edu.gh (A.-A.S.); 3 College of Public Health, Medical and Veterinary Services, James Cook University, Townsville, QLD 4811, Australia; 4 Department of Health, Physical Education, and Recreation, University of Cape Coast, PMB TF0494 Cape Coast, Ghana; 5 Neurocognition and Action-Biomechanics-Research Group, Faculty of Psychology and Sports, Science, Bielefeld University, Postfach 10 01 31, 33501 Bielefeld, Germany; 6 School of Public Health, Faculty of Health, University of Technology Sydney, Sydney, NSW 2007, Australia; brightahinkorah@gmail.com

**Keywords:** attitudes, intimate partner violence, public health, sub-Saharan Africa, women

## Abstract

Intimate partner violence (IPV) is predominant in sub-Saharan Africa (SSA), with nearly 40 percent of women reporting IPV at some point in time. In this study, we investigated whether a supportive attitude towards IPV is associated with past-year experience of IPV among women in sexual unions in SSA. This study involved a cross-sectional analysis of data from the Demographic and Health Survey (DHS) of 23 countries in SSA. Bivariate and multivariable binary logistic regression analyses were performed to determine the association between attitude towards IPV and past-year experience of IPV. The regression results were presented in a tabular form using crude odds ratio (cOR) and adjusted odds ratio (aOR) at 95% confidence intervals (CIs). In the pooled countries, we found that women who had supportive attitude towards IPV were more likely to experience IPV compared to those who rejected IPV (cOR = 1.72, 95% CI = 1.64, 1.79), and this persisted after controlling for maternal age, marital status, wealth, maternal education level, place of residence, and mass-media exposure (aOR = 1.72, 95% CI = 1.64, 1.79). The same trend and direction of association between attitude towards IPV and experience of IPV was also found in all the 23 studied countries. This study has demonstrated that women who accept IPV are more likely to experience IPV. Hence, we recommend that efforts to end IPV must focus primarily on changing the attitudes of women. This goal can be achieved by augmenting women’s empowerment, education, and employment interventions, as well as sensitizing women in relation to the deleterious ramifications of accepting IPV. Furthermore, reducing IPV is critical towards the achievement of Sustainable Development Goal 3.

## 1. Introduction

Intimate partner violence (IPV) is a pervasive social injustice and significant public health concern [1,2,3]. Available evidence suggests that IPV, which refers to any behavior within an intimate relationship that inflicts physical, psychological, and sexual harm [4], can be regarded as a pandemic, as one in every three women in the world have experienced IPV at some point in their lives [5]. Nonetheless, IPV is predominant in sub-Saharan Africa (SSA), with nearly 40 percent of women who have ever been in an intimate relationship having reported IPV at some point in time [6]. Although IPV can be perpetrated by anyone, there is compelling evidence to show that men are usually the culprits, while women are usually the victims [7,8].

The negative effects of IPV cannot be understated. It is a clear violation of the rights of women and has been shown to be significantly associated with several adverse health outcomes among women [9,10,11,12]. Women who experience IPV have been found to suffer numerous negative mental-health outcomes including low self-esteem, anxiety, post-traumatic stress syndrome, and depression [13]. Moreover, there is the tendency for women who experience IPV to have suicidal ideation and attempt [14].

Preventing and mitigating IPV against women has thus become a critical public-health discourse, and this is reflected by its inclusion in the Sustainable Development Goals (SDGs) [15,16]. The existing body of knowledge on IPV indicates that inequalities and disparities in the socio-economic status quo of women can prevent or exacerbate the likelihood of their experiencing IPV [9,10,11,12,17,18,19]. Factors such as age, marital status, mass-media exposure, educational attainment, and employment status of women have been found to be significant in predicting the chances of a woman experiencing IPV [9,10,11,12,17]. However, beyond these personal characteristics and socio-economic conditions, could attitude towards IPV be a precursor of experiencing IPV? This question remains unanswered in existing IPV studies. There is an increasing belief that a supportive attitude towards IPV may reinforce and exacerbate the likelihood of experiencing IPV [18,19]. Notwithstanding, previous studies have centered on attitudes towards IPV [18,19] but have not examined how such attitudes are associated with the experience of IPV. Therefore, we sought to investigate whether a supportive attitude towards IPV was associated with past-year experience of IPV among women in SSA. We hypothesized that women with a supportive attitude towards IPV are more likely to experience IPV than those without supportive attitude towards it. The present study responds to the rising need for empirical evidence to strengthen Africa’s response to IPV.

## 2. Materials and Methods

### 2.1. Data Source and Study Design

This present study involved a cross-sectional analysis of data from the Demographic and Health Survey (DHS) of23 countries in SSA (see Table 1). The DHS is a nationally representative survey carried out in over 85 low-and-middle-income countries since its inception in 1984 [20]. It collects a wide range of objective and self-reported data, with a strong focus on indicators such as fertility, reproductive health, maternal and child health, mortality, nutrition, and self-reported health behaviors among adults [20]. The DHS adopts a two-stage stratified sampling technique to collect the nationally representative data from the respondents, with a detailed explanation of the sampling procedure provided in a study by Aliaga and Ruilin [21]. The inclusion criteria for the selection of the 23 countries were countries that had their most recent DHS published between 2010 and 2019 and also had the domestic violence module, which contained questions on IPV. Hence, countries were excluded if their most recent DHS was published before 2010 and did not contain the domestic violence module. A total of 100,182 married and cohabiting women aged 15–49 years with complete cases of the variables of interest were extracted and included in the final analysis (Table 1). A detailed description of the sample extracted for the study can be found in Table 1. The dataset is freely available for download at https://dhsprogram.com/data/available-datasets.cfm (accessed on 21 January 2021). We relied on the “Strengthening the Reporting of Observational Studies in Epidemiology” (STROBE) statement in writing the manuscript [22].

### 2.2. Study Variables

#### 2.2.1. Outcome Variable

The outcome variable was past-year experience of IPV. This was assessed using three key variables (physical violence [PV], emotional violence [EV], and sexual violence [SV]). These variables were derived from the optional domestic violence module, where questions are based on a modified version of the conflict tactics scale [23,24]. The questions used to measure physical violence in the 12 months preceding the survey were whether the respondent’s partner ever: pushed, shook, or threw something at her; slapped her; struck her with his fist or something harmful; kicked or dragged her; strangled or burnt her; threatened her with a knife, gun, or other weapon; and twisted her arm or pulled her hair. Questions on emotional violence include whether her partner ever: humiliated her; threatened to harm her; and insulted or made her feel bad. Sexual violence questions were whether the partner ever: physically forced the respondent into unwanted sex; forced her into other unwanted sexual acts; and physically forced her to perform sexual acts she did not want to perform. The response options in each of the questions were “never”, “often”, “sometimes”, and “yes, but not in the last 12 months”. For this study’s purpose, the responses were dichotomized into “No” (those who responded as never and yes, but not in the last 12 months) and “Yes” (those who responded as often and sometimes). Later, an index variable called the IPV in the past 12 months was created using the dichotomized responses for physical, emotional, and sexual violence. A woman was considered to have experienced IPV if she had experienced any of the three key variables (physical violence, emotional violence, and/or sexual violence).

#### 2.2.2. Explanatory Variable

The explanatory variable was attitude towards violence. A total of five items were used to measure this variable. All women in sexual unions were asked if their husbands/partners were justified for wife-beating for the following reasons: (i) burning food, (ii) arguing with him, (iii) going out without telling him, (iv) neglecting the children, and (v) refusing to have sexual intercourse with him. The response options were “no”, “yes”, and “don’t know”. To help create the attitude towards violence variable, all those who responded “no” were classified as “rejecting their husbands/partners’ justification for wife-beating in at least one of the five reasons”, while those who responded “yes” were considered as “supportive of husband’s/partner’s justification for wife-beating”. The selection of the items to measure the attitude towards violence was informed by literature [25].

#### 2.2.3. Covariates

The covariates included in our study were selected based on their significant associations with IPV [25,26,27,28] and also their availability in the DHS dataset. The covariates include maternal age, marital status, wealth, maternal education level, place of residence, and mass-media exposure. We used the existing DHS coding for maternal age, wealth, and place of residence. Marital status was recoded as “married” and “cohabiting”. Maternal education level coded in the DHS dataset as no education, primary, secondary, and higher was recoded as “no education”, “primary”, and “secondary or higher”. Exposure to media was created from three variables (frequency of watching television, frequency of reading newspaper/magazine, and frequency of listening to the radio). Each of the variables has the same response options: not at all, less than once a week, at least once a week, and almost every day. The responses were recoded as “No = not at all” and “Yes” (less than once a week, at least once a week, and almost every day). Lastly, a third variable named mass media was created. Any woman with exposure to at least one of these (watching television, reading newspaper/magazine, and listening to the radio) was said to have mass-media exposure.

### 2.3. Statistical Analyses

**Figure 1:**
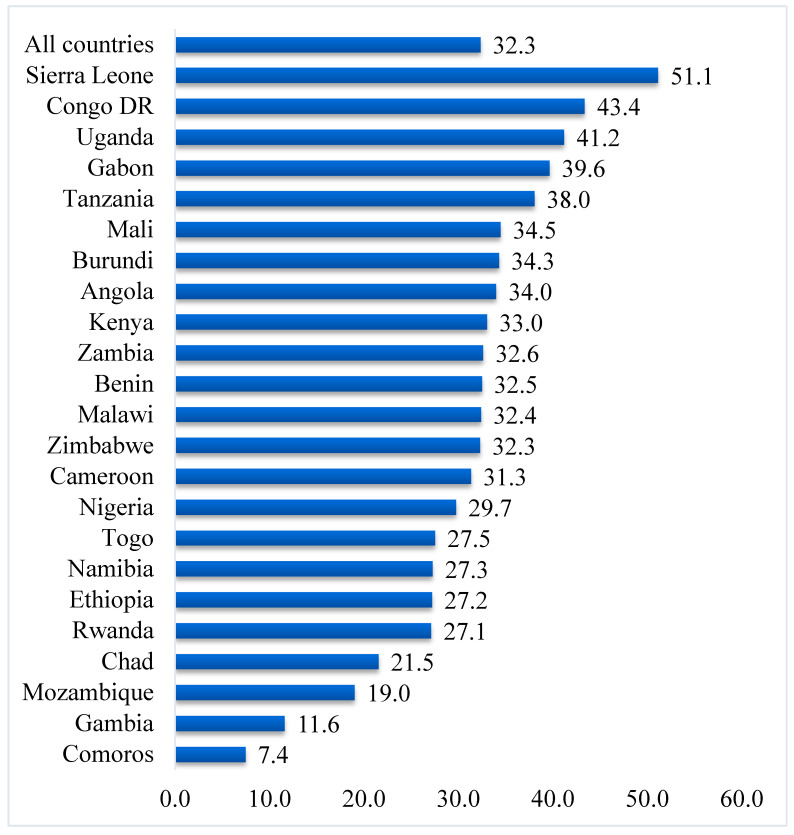
Prevalence of past-year experience of intimate partner violence (%).

**Figure 2:**
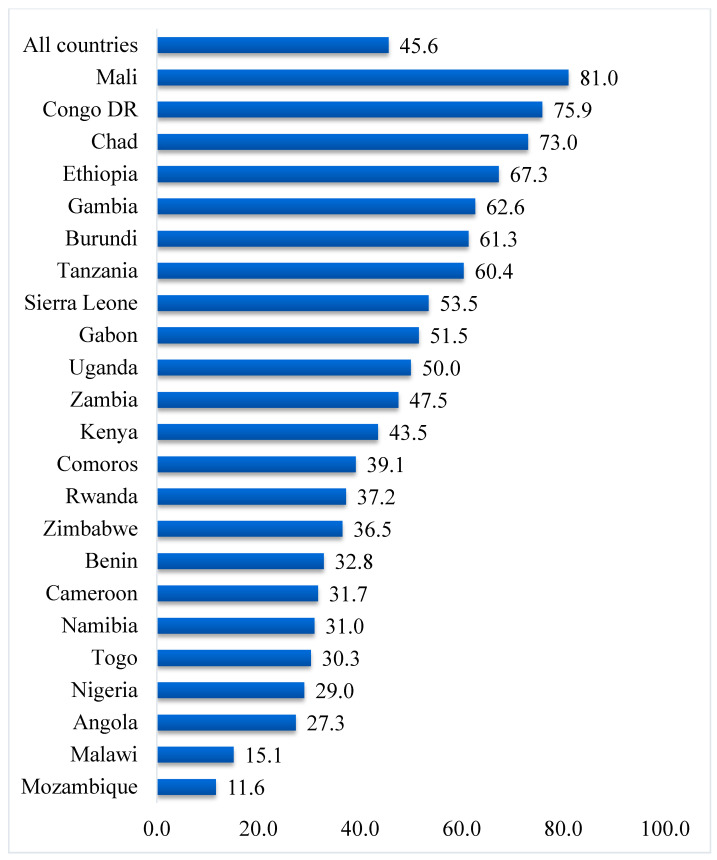
Prevalence of supportive attitude towards intimate partner violence (%).

Data were analyzed using Stata version 16.0 (Stata Corporation, College Station, TX, USA). The analyses were carried out in three stages. Firstly, the proportions of past-year experience of IPV and attitude towards IPV were presentedas shown in Figure 1 and Figure 2, respectively. Secondly, a Pearson’s chi-square test was conducted to determine the distribution of attitude towards past-year experience of physical, emotional, sexual, and IPV across the 23 SSA countries. The results of the chi-square analyses were also used to determine the relationship between attitude towards IPV and past-year experience of IPV. Next, bivariate and multivariable binary logistic regression analyses were performed to determine the effect of attitude towards IPV and past-year experience of IPV in SSA. The regression results were presented in a tabular form using crude odds ratio (cOR) and adjusted odds ratio (aOR) at 95% confidence intervals (CIs). Statistical significance was set at *p* < 0.05 in the chi-square test and regression analysis. The women’s sample weights for the domestic violence module (d005/1,000,000) were applied to obtain unbiased estimates, according to the DHS guidelines, and the survey command (SVY) in Stata was used to adjust for the complex sampling structure of the data in the regression analyses. A multicollinearity test was conducted using the variance inflation factor (VIF) and the results showed no evidence of multicollinearity among the variables studied.

### 2.4. Ethical Approval

Ethical clearances were obtained from the Ethics Committee of the ORC Macro Inc and the ICF Institutional Review Board in participating countries. The ICF IRB ensured that the survey complied with the U.S. Department of Health and Human Services regulations for the protection of human subjects, while the participating country’s IRB ensured that the survey complied with the laws and norms of the nation. All the ethical guidelines regarding the conduct of studies using humans were strictly adhered to. This was a secondary analysis of data, and therefore no further approval was required since the data are available in the public domain. Detailed information about the DHS data usage and ethical standards are available at http://goo.gl/ny8T6X, accessed on 1 March 2021.

## 3. Results

### 3.1. Prevalence of Supportive Attitude for IPV and Past-Year Experience of IPV

The proportion of women who had experienced IPV in the year preceding the survey in the 23 countries was 32.3%, ranging from as high as 51.1% in Sierra Leone to as low as 7.4% in Comoros (Figure 1). In total, 45.6% of women in the 23 countries had a supportive attitude towards IPV; the highest prevalence was recorded in Mali (81.0%), while the lowest prevalence was found in Mozambique (11.6%) (Figure 2).

### 3.2. Distribution and Attitude Across IPV among Women

Table 2 presents results on the distribution of attitude towards IPV across past-year experience of physical, emotional, sexual, and intimate partner violence by country. In all 23 countries, the prevalence of PV, EV, SV, and IPV were significantly higher among women who had a supportive attitude towards PV (24.2%), EV (27.6%), SV (12.6%), and IPV (38.8%) than those who rejected them (14.6%, 20.6%, 6.6%, and 26.9%, respectively). In terms of country-specific results, except in Cameroon and Comoros, PV was significantly higher among women who had a supportive attitude towards IPV in all the countries compared to those who rejected PV. EV was also significantly higher among women who had a supportive attitude towards it compared to those who rejected it in all the countries except Chad, Benin, Comoros, and Ethiopia. With SV, women who had a supportive attitude towards it were more prevalent than those who rejected it in all the countries apart from Cameroon, Chad, Gambia, and Rwanda. The prevalence of IPV was higher among women who had a supportive attitude towards IPV than those who rejected it in all the countries, except Comoros.

### 3.3. Association between Attitude towards IPV and Past-Year Experience of IPV

Table 3 presents the results of the association between attitude towards IPV and past-year experience of IPV. In the pooled results, we found that women who had a supportive attitude towards IPV were more likely to experience IPV compared to those who rejected IPV (cOR = 1.72, 95% CI = 1.64–1.79), and this persisted after controlling for maternal age, marital status, wealth, maternal education level, place of residence, and mass-media exposure (aOR = 1.72, 95% CI = 1.64–1.79). The same trend and direction of association between attitude towards IPV and experience of IPV was also found in all the 23 countries considered in this study.

## 4. Discussion

IPV has been a perennial social injustice that cuts across different populations and socio-cultural contexts [2,3]. Hence, it has gained the attention of researchers in recent times. Although there are several studies on IPV in Africa, the focus has not been on the extent to which supportive attitudes contribute to the likelihood of experiencing the phenomenon. Therefore, the study examined whether a supportive attitude was associated with experiencing IPV, using data from the DHS of 23 countries in SSA.

Findings show that, on average, 3 out of 10 women had experienced IPV within the 12 months prior to the survey. However, the prevalence of past-year IPV experience varied among the different countries, with Sierra Leone recording the highest prevalence and Comoros reporting the lowest prevalence. This outcome supports the findings of Izugbara et al. [17], who also reported Comorian women to be safest in terms of IPV experience, and Sierra Leone to be most unsafe. This trend is probably due to the many conflicts that have ravaged Sierra Leone for years. In times of conflict, social-protection systems drastically deteriorate, thereby exacerbating other risk factors and exposing more women to IPV [29,30]. Regarding supportive attitudes towards IPV, Mali reported the highest prevalence, whereas Mozambique reported the lowest prevalence.

It was also observed that supportive attitudes towards IPV increased the likelihood of experiencing IPV in the past year by 1.7-fold, even after controlling for maternal age, marital status, wealth, maternal education level, place of residence, and mass-media exposure. This result corroborates other studies in Africa and low-and middle-income countries in general [18,19]. A plausible explanation for this finding is that the patriarchal norms and beliefs, coupled with sustained community tolerance for IPV could make women believe that their intimate partners can abuse them under certain conditions [31,32]. Moreover, a report from Ghana has shown that women’s approval of IPV prevents them from reporting an incidence of IPV when it occurs, and subsequently increases their odds of experiencing IPV in the past year [33]. Alternatively, supportive attitudes towards IPV lead to the internalization of IPV, and subsequently make it a risk factor for later perpetration as well as relational and overt victimization [34,35].

### Strength and Limitations

The strength of our study is in the use of a nationally representative survey dataset which increases the generalizability of the findings to women from the selected sub-Saharan African countries. Notwithstanding this strength, the use of cross-sectional data limits the inferences that can be made from the findings. At best, only associations can be inferred from this study, and not causality. Hence, interpretations of findings must be noted with caution. Furthermore, the findings of the present study may not necessarily be reflective of other African countries and non-African countries that were not included in this study. Nevertheless, current findings provide evidence of the role of supportive attitudes towards IPV in predicting the likelihood of experiencing IPV. As such, stakeholders can leverage on the findings to design and effectively implement policies as well as appropriate interventions that seek to reorient women and eliminate the supportive attitudes that facilitate the tendency to experience IPV. In terms of future perspectives, it would be interesting to test these results in non-African countries.

## 5. Conclusions

The study has demonstrated that women who justifyIPV are more likely to experience IPV. Hence, we recommend that efforts to end IPV should focus on changing women’s attitudes. This goal can be achieved by augmenting women’s empowerment, education, and employment interventions, as well as the sensitization of women in relation to the deleterious ramifications of accepting IPV.

## Figures and Tables

**Table 1:** Description of sample.

Countries	Year of Survey	Weighted Sample	Weighted Percentage
Central Africa			
Angola	2015–2016	8271	8.3
Cameroon	2018	4003	4.0
Chad	2014–2015	3444	3.4
Congo DR	2013–2014	4754	4.7
Gabon	2012	3043	3.0
West Africa			
Benin	2018	4180	4.2
Gambia	2013	3243	3.2
Mali	2018	3415	3.4
Nigeria	2018	8562	8.5
Sierra Leone	2019	3663	3.7
Togo	2013–2014	4796	4.8
East Africa			
Burundi	2016–17	6057	6.0
Comoros	2012	2160	2.2
Ethiopia	2016		
Kenya	2014	3587	3.6
Mozambique	2011	2322	2.3
Rwanda	2014–2015	1518	1.5
Tanzania	2015–2016	6409	6.4
Uganda	2016	6157	6.1
Southern Africa			
Malawi	2015–2016	4551	4.5
Namibia	2013	910	0.9
Zambia	2018	5874	5.9
Zimbabwe	2015	5011	5.0
All countries		100,182	100.0

**Table 2:** Attitude towards IPV and past-year experience of IPV among women in sub-Saharan Africa by country.

Countries	Past-Year Experience of PV		*p*-Values	Past-Year Experience of EV		*p*-Values	Past-Year Experience of SV		*p*-Values	Past-Year Experience of IPV		*p*-Values
	Rejection Attitude	Supportive Attitude		Rejection Attitude	Supportive Attitude		Rejection Attitude	Supportive Attitude		Rejection Attitude	Supportive Attitude	
All countries	14.6	24.2	<0.001	20.6	27.6	<0.001	6.6	12.6	<0.001	26.9	38.8	<0.001
Central Africa												
Angola	19.1	37.3	<0.001	21.0	31.6	<0.001	4.6	12.1	<0.001	28.9	47.4	<0.001
Cameroon	18.3	21.2	0.131	20.2	27.0	0.001	6.3	7.6	0.352	29.0	36.4	0.001
Chad	9.6	15.9	0.001	12.9	15.6	0.272	6.0	6.5	0.703	16.8	23.2	0.012
Congo DR	17.7	33.1	<0.001	21.6	30.6	<0.001	13.3	21.3	<0.001	31.5	47.1	<0.001
Gabon	20.8	35.0	<0.001	21.9	29.6	0.010	7.4	14.3	<0.001	31.7	47.1	<0.001
West Africa												
Benin	8.2	16.5	<0.001	28.7	30.6	0.379	4.9	8.4	0.001	30.8	35.8	0.022
Gambia	2.6	8.4	<0.001	5.8	9.3	0.022	0.8	1.2	0.423	7.5	14.0	<0.001
Mali	12.9	19.5	0.019	20.2	30.3	<0.001	4.2	8.9	0.002	23.8	37.0	<0.001
Nigeria	10.3	14.4	<0.001	24.8	32.3	<0.001	3.9	6.1	0.001	26.9	36.6	<0.001
Sierra Leone	33.2	44.1	<0.001	34.5	43.0	<0.001	5.0	7.3	0.030	45.6	55.9	<0.001
Togo	8.2	14.5	<0.001	22.3	30.5	<0.001	3.8	6.3	0.001	24.5	34.4	<0.001
East Africa												
Burundi	12.9	22.9	<0.001	11.5	20.9	<0.001	13.4	24.7	<0.001	24.2	40.7	<0.001
Comoros	3.0	4.6	0.129	5.5	6.0	0.722	0.5	1.8	0.013	6.2	9.3	0.073
Ethiopia	14.2	18.1	0.036	17.8	21.3	0.115	4.6	10.5	<0.001	22.2	29.6	0.001
Kenya	18.4	27.4	<0.001	19.6	29.6	<0.001	7.7	12.1	0.001	28.1	39.4	<0.001
Mozambique	12.8	23.7	<0.001	11.0	18.2	0.010	2.0	4.9	0.040	17.4	31.4	<0.001
Rwanda	14.8	21.2	0.002	15.4	24.2	<0.001	7.1	10.3	0.143	22.4	35.0	<0.001
Tanzania	18.5	31.9	<0.001	20.9	32.8	<0.001	6.9	11.5	<0.001	28.2	44.5	<0.001
Uganda	17.5	27.9	<0.001	26.0	35.2	<0.001	12.4	21.0	<0.001	33.7	48.6	<0.001
Southern Africa												
Malawi	14.6	20.0	0.010	21.0	30.3	<0.001	14.3	22.0	<0.001	30.3	44.3	<0.001
Namibia	13.9	27.6	<0.001	17.0	27.5	0.001	4.0	10.7	0.001	21.7	39.7	<0.001
Zambia	14.7	27.5	<0.001	17.1	28.1	<0.001	6.1	16.5	<0.001	24.0	42.2	<0.001
Zimbabwe	14.3	18.7	0.001	23.1	28.7	0.001	7.8	12.6	<0.001	28.9	38.1	<0.001

Note: Pearson chi-square test was used to obtain *p*-values; PV = Physical violence; EV = Emotional violence; SV = Sexual violence; IPV = Intimate partner violence.

**Table 3:** Logistic regression on the association between attitude towards IPV and past-year experience of IPV in sub-Saharan Africa.

Countries	Model I	Model II
	cOR (95%CI)	aOR (95%CI)
All countries	1.72 *** (1.64, 1.79)	1.72 *** (1.64–1.79)
Central Africa		
Angola	2.54 *** (2.28, 2.84)	2.63 *** (2.34, 2.94)
Cameroon	1.67 *** (1.44, 1.92)	1.71 *** (1.47, 1.98)
Chad	1.83 *** (1.49, 2.25)	1.72 *** (1.39, 2.12)
Congo DR	2.32 *** (2.03, 2.67)	2.29 *** (1.99, 2.63)
Gabon	1.99 *** (1.73, 2.29)	1.94 *** (1.68, 2.24)
West Africa		
Benin	1.27 ** (1.11, 1.46)	1.20 * (1.04, 1.38)
Gambia	1.98 *** (1.57, 2.50)	2.34 *** (1.83, 2.98)
Mali	2.32 *** (1.91, 2.82)	2.27 *** (1.86, 2.78)
Nigeria	1.66 *** (1.50, 1.82)	1.57 *** (1.41, 1.75)
Sierra Leone	1.67 *** (1.47, 1.90)	1.69 *** (1.45, 1.92)
Togo	1.74 *** (1.53, 1.97)	1.52 *** (1.33, 1.73)
East Africa		
Burundi	2.14 *** (1.91, 2.39)	1.97 *** (1.76, 2.21)
Comoros	1.59 ** (1.17, 2.15)	1.58 ** (1.16, 2.16)
Ethiopia	1.44 *** (1.24, 1.67)	1.34 *** (1.14, 1.57)
Kenya	1.65 *** (1.43, 1.89)	1.61 *** (1.39, 1.85)
Mozambique	1.85 *** (1.38, 2.47)	1.95 *** (1.44, 2.64)
Rwanda	1.71 *** (1.36, 2.13)	1.63 *** (1.29, 2.06)
Tanzania	2.28 *** (2.04, 2.54)	2.08 *** (1.85, 2.32)
Uganda	1.96 *** (1.77, 2.17)	1.80 *** (1.62, 1.99)
Southern Africa		
Malawi	1.73 *** (1.47, 2.04)	1.70 *** (1.44, 2.02)
Namibia	2.08 *** (1.61, 2.70)	1.81 *** (1.37, 2.39)
Zambia	2.16 *** (1.93, 2.41)	2.03 *** (1.82, 2.28)
Zimbabwe	1.55 *** (1.37, 1.76)	1.46 *** (1.29, 1.67)

Model 1: Unadjusted model examining the independent association between attitude towards IPV and past-year experience of IPV. Model 2: Adjusted for maternal age, marital status, wealth, maternal education level, place of residence, and mass-media exposure. cOR is the crude odds ratio; aOR is the adjusted odds ratio. Reference categories were those who rejected IPV; * *p* < 0.05; ** *p* < 0.01; *** *p* < 0.001.

## Data Availability

The dataset is freely available for download at https://dhsprogram.com/data/available-datasets.cfm (accessed on 21 January 2021).
